# Ophthalmomyiasis in a preterm neonate resulting in blindness: A case report from Botswana

**DOI:** 10.3389/fped.2022.955212

**Published:** 2022-09-29

**Authors:** Britt Nakstad, Yeni Zandile, Kesiilwe Gaebolae, Francis Msume Banda, Tebo Dinotshe, Fizzah Imran, Alemayehu Mekonnen Gezmu

**Affiliations:** ^1^Department of Paediatrics and Adolescent Health, University of Botswana, Gaborone, Botswana; ^2^Division Paediatric Adolescent Medicine, Inst Clinical Medicine, Faculty of Medicine, University of Oslo, Oslo, Norway; ^3^Department of Ophthalmology, Princess Marina Hospital, Gaborone, Botswana

**Keywords:** preterm neonate, ophthalmomyiasis, blindness, jaundice, eye-covering during phototherpy

## Abstract

Myiasis is an infestation of human tissue by insect larvae. While rare, healthcare-associated myiasis has been reported from immobilized patients in resource-limited healthcare facilities in warm climates without adequate vector control measures. We describe a case of Ophthalmomyiasis in a hospitalized neonate in Botswana that resulted in vision loss. The neonate, who was initially hospitalized due to the complications of prematurity, received phototherapy for jaundice, and to avoid phototherapy-related retinopathy, the neonate’s eyes were covered using cotton gauze and adhesive tapes that potentially damaged the skin as commercially available eye covering was not in stock. Therefore, eye covering was not changed and when the eye covering was removed almost 3 days after placement, insect larvae were noted in the patient’s eyes and nose. Ophthalmologic evaluation revealed perforated corneal ulcer and uveal prolapse in the right eye resulting in complete blindness and corneal scarring of the left eye. The patient’s clinical course was further complicated by an *Enterobacter* species bloodstream infection. This case highlights the importance of vector control as a major patient safety measure for neonatal units in warm climates. Flies had been observed in the room and mitigation measures included reducing fly populations through traps, screens, and removal of standing water and leftover food. Every mother and staff were sanitizing hands when entering the room and gowns were used. This case also reinforces the importance to conduct vigilant monitoring of patients, especially neonates with eyes covered during phototherapy.

## Introduction

Myiasis is an infestation of human tissue by larvae of a variety of fly species. Insect larvae (i.e., maggots) feed off and develop in the tissues of living organisms when adult flies lay eggs in or on the tissues. While rare, myiasis has been reported in adults living in tropical and subtropical regions, particularly as wound infestations in settings where flies are present and are able to contact openings and wounds (1, 2). Healthcare-associated myiasis is associated with insufficient supervision of immobilized patients in warm climates (3). Myiasis is even rare in neonates. Whereas most published cases in neonates describe community-acquired umbilical myiasis (4–10), neonatal ophthalmomyiasis has been scantily described (2, 11, 12). Other sites reported among neonates are ear (13), nasopharynx (14), oral (15), skin (16), and intestine (17). Powdered infant formula has also been noted to have been contaminated (18). A case of neonatal myiasis leading to *Enterobacter cloacae* sepsis was described in a premature neonate in Botswana (not reported), but to our knowledge, this is the first report of neonatal ophthalmomyiasis leading to blindness. This case report refers to a vulnerable neonate born 13 weeks before term with very low birth weight, needing intensive care and follow-up, and cared for in an intensive care unit that was understaffed, overcrowded, and equipment and reagents for measuring hyperbilirubinemia out of stock. This paved the way for improved guidelines outlining frequent (every 8 h) checks of eye coverings and implementation of vector control as an infection prevention measure. Although this unit is staffed by competent and caring healthcare personnel, this report reminds us that staff shortages can compromise the safety, especially in healthcare settings prone to insect infestation.

## Case description

The parents of this patient provided written consent for the data collection and presentation of findings.

### Medical history

In Botswana during the summer months, a male neonate was born to an HIV-negative and venereal research laboratory (VDRL) non-reactive mother at 27 weeks gestational age (GA). APGAR scores were 5/7/8 at first/fifth/10th minutes, respectively, and he required resuscitation by mask and bag ventilation at birth. He was given tetracycline eye ointment which is routinely given as prophylaxis against ophthalmia neonatorum as delivering mothers in Botswana are not screened for chlamydia and gonococcal infections. Vitamin K was given intramuscularly to prevent hemorrhagic disease. Due to persistent respiratory distress, the patient was admitted to the neonatal unit of a tertiary hospital, intubated, mechanically ventilated, and started on first-line antibiotics (ampicillin and gentamicin) as empiric treatment for suspected sepsis. Initial white blood cell counts were 11.87 × 10^9^/L (26% neutrophils) and platelets 207 × 10^9^/L. Infection biomarkers were not available, but the blood culture collected prior to empiric antibiotic administration grew *Enterobacter* sp. (un-speciated).

The patient was noted to be clinically jaundiced age 30 h (serum total bilirubin was 184 mmol/L), secondary to suspected Rh isoimmunization, and was started on phototherapy (PT) and intravenous immunoglobulin. To prevent PT-associated retinopathy, the patient’s eyes were covered using cotton gauze and adhesive tape because commercially available eye covering was not in stock.

On day-of-life (DOL) 2–4, the patient displayed signs of clinical deterioration: pulmonary hemorrhage (as evidenced by fresh blood in the endotracheal tube, treated with fresh frozen plasma, vitamin K, and adrenaline via the endotracheal tube), Grade 3 intraventricular hemorrhage, and acute kidney injury (urea 14.1 mmol/L and creatinine 89 mmol/L). Antibiotic coverage was broadened to piperacillin–tazobactam and amikacin.

**FIGURE 1:**
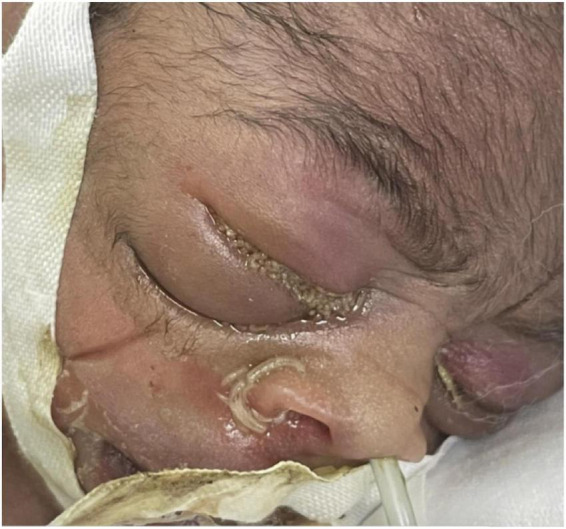
Insect larvae in the eyes and coming out of the nostrils.

Because the neonatal unit only had cotton gauze and adhesive tape for use as an eye covering, there was a fear to damage the skin if the adhesive tape was repeatedly removed during phototherapy. However, when the patient’s serum bilirubin had declined to below phototherapy indication levels at DOL3, the eye covering was removed. There was now bilateral periorbital edema with erythema, the right eye being more affected than the left. Insect larvae of different sizes were identified in both eyes upon retracting the eyelids and some were even seen coming out of the nostrils. None were noted in from the mouth or auricular orifices (see Figure 1).

**FIGURE 2:**
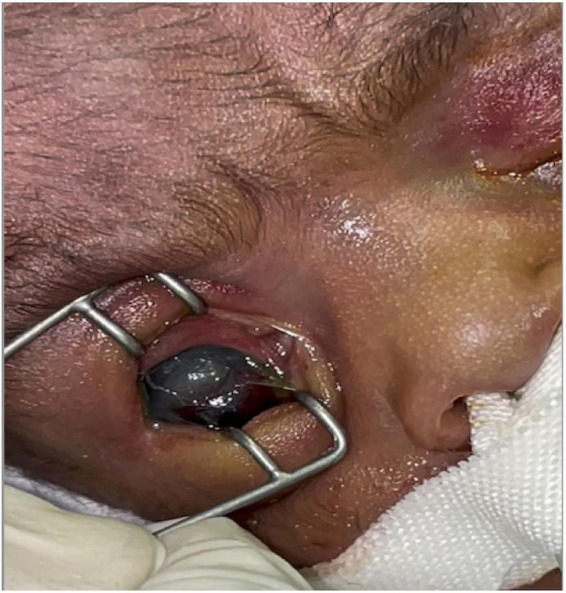
The right eye perforated corneal ulcer with uveal prolapse.

Insect larvae were immediately and carefully removed manually, followed by rinsing the eyes with sterile, normal saline. Chloramphenicol eye ointment was initially applied to both eyes. Upon ophthalmologist’s review, it was discovered that the patient had a corneal ulcer in the left eye with injected and reddish conjunctival tissue, while deeper structures were difficult to explore due to a hazy cornea. There was periorbital swelling and the conjunctival and surrounding tissues were injected and inflamed. In the right eye, a perforated corneal ulcer with uveal prolapse was observed on the inferior aspect of the cornea (Figure 2). The left eye was lavaged two to three times daily with sterile saline to wash out pus. Topical tetracycline plus gentamicin eye ointment and ofloxacin eye drops were alternated 2 hourly. According to the ophthalmologist, evisceration of the right eye could be done once the baby was off the ventilator and become clinically stable.

**FIGURE 3:**
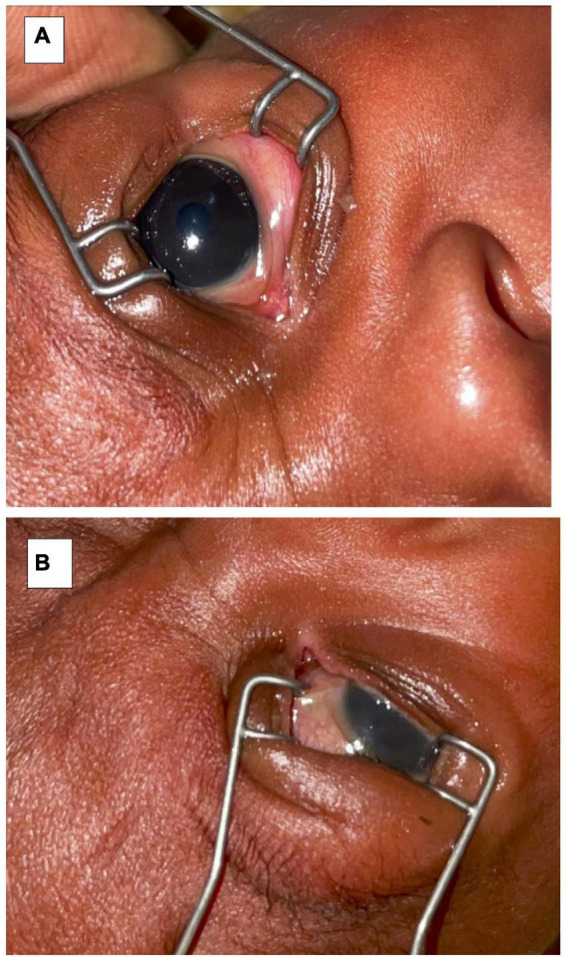
Upon discharge **(A)** left eye examination showing a corneal scar and **(B)** right eye showing phthisis bulbi and symblepharon.

On discharge, the corneal ulcer on the left eye had healed with a corneal scar. There was right eye phthisis bulbi (Grade I) with a symblepharon inferiorly. Evisceration was, therefore, deemed no longer necessary (Figure 3).

There were no signs of retinopathy of prematurity (ROP) screening on further ophthalmologic assessment of the left eye. The retina was fully vascularized up to the far periphery (Stage 0 ROP, Zone III). The patient was discharged to his local hospital on topical artificial tears four times a day on the right eye only, and to continue follow-up in ophthalmology clinic as an outpatient.

## Discussion

We report a case of neonatal ophthalmomyiasis which was likely delayed in being diagnosed due to an eye covering *in situ* during phototherapy. This led to complete blindness in one eye, while the other eye healed well and resulted in a corneal scar only. This case emphasizes the vulnerabilities of hospitalized neonates in inpatient units that periodically experience insect infestations.

This case highlights the importance of vigilant monitoring of neonates as they are unable to mechanically remove flies or maggots from their faces, including eyes. Understaffed units may not be equipped to monitor patients with the frequency and thoroughness which is needed to detect such abnormalities. Sick and hospitalized neonates may be especially at risk and predisposed to insect infestation in covered eyes, wounds, and umbilical stumps if flies are in the room as they often are kept naked under a radiant warmer, for observation. Prematurity may also pose an increased risk of infection due to an immature immune system.

Treatment of myiasis consists of removal of the larvae and control of local and systemic infection, if any. In our case, any dead or decaying deep-seated larvae that could cause secondary infection or sepsis were removed manually, followed by daily lavage with sterile normal saline, local treatment with antimicrobial ointments, and continuous intravenous antibiotic treatment. Petroleum ointment or topical ivermectin solution may be an alternative medication to smoothly remove or kill larvae (19). Surgery was not necessary or indicated. The fact that this is the second case of *Enterobacter* sepsis following neonatal myiasis in this setting emphasizes the potential of flies and fly larvae to serve as transmission vehicles for bacterial infection (20).

This neonate was subjected to several hours of eye covering using cotton and tape which was the only available material. There was a fear to damage the skin if the adhesive tape was repeatedly removed during phototherapy. The skin and ocular area under the covering were not examined during phototherapy, not until treatment was stopped (Figure 1). According to the neonatal nursing staff, his eyes had been continuously covered and the tape removed intermittently to avoid traumatizing the skin in the face. The routine for eye covering in our unit has now been changed and procedures introduced to check the area under the eye covering two times daily when a neonate is under phototherapy.

### Essential points and patient perspective

Neonatal myiasis is a risk in warm climates, particularly among neonates with covered eyes during phototherapy for neonatal jaundice, open wounds, or an exposed umbilical stump. Neonatal units must include vector control as a core patient safety strategy. Mitigation measures include reducing fly populations through traps, screens, and removal of standing water and food. Additionally, adequate staffing and access to non-adhesive eye coverings are essential for healthcare workers to complete frequent and thorough monitoring.

## Data availability statement

The raw data supporting the conclusions of this article will be made available by the authors, without undue reservation.

## Ethics statement

Ethical review and approval was not required for the study on human participants in accordance with the local legislation and institutional requirements. Written informed consent to participate in this study was provided by the participants’ legal guardian/next of kin. Written informed consent was obtained from the individual(s), and minor(s)’ legal guardian/next of kin, for the publication of any potentially identifiable images or data included in this article.

## Author contributions

BN drafted the manuscript with inputs and acceptance of the final version of the manuscript by all co-authors. All authors were involved in treatment and discussions related to the patient and accepted the final version of the manuscript.
